# Footwear comfort: a systematic search and narrative synthesis of the literature

**DOI:** 10.1186/s13047-021-00500-9

**Published:** 2021-12-07

**Authors:** Hylton B. Menz, Daniel R. Bonanno

**Affiliations:** Discipline of Podiatry, School of Allied Health, Human Services and Sport, La Trobe University, Melbourne, Victoria 3086 Australia

**Keywords:** Shoes, Foot orthoses, Comfort, Measurement

## Abstract

**Objective:**

To provide a narrative synthesis of the research literature pertaining to footwear comfort, including definitions, measurement scales, footwear design features, and physiological and psychological factors.

**Methods:**

A systematic search was conducted which yielded 101 manuscripts. The most relevant manuscripts were selected based on the predetermined subheadings of the review (definitions, measurement scales, footwear design features, and physiological and psychological factors). A narrative synthesis of the findings of the included studies was undertaken.

**Results:**

The available evidence is highly fragmented and incorporates a wide range of study designs, participants, and assessment approaches, making it challenging to draw strong conclusions or implications for clinical practice. However, it can be broadly concluded that (i) simple visual analog scales may provide a reliable overall assessment of comfort, (ii) well-fitted, lightweight shoes with soft midsoles and curved rocker-soles are generally perceived to be most comfortable, and (iii) the influence of sole flexibility, shoe microclimate and insoles is less clear and likely to be more specific to the population, setting and task being performed.

**Conclusion:**

Footwear comfort is a complex and multifaceted concept that is influenced not only by structural and functional aspects of shoe design, but also task requirements and anatomical and physiological differences between individuals. Further research is required to delineate the contribution of specific shoe features more clearly, and to better understand the interaction between footwear features and individual physiological attributes.

## Background

Footwear plays an essential role in protecting the foot from trauma and facilitating efficient and pain-free movement when performing a wide range of routine, occupational, recreational, and sporting activities. The selection of footwear is influenced by economic, cultural and functional factors, with comfort frequently being reported as one of the most important considerations in a range of settings [1–3]. Comfort can be defined as the state of being physically relaxed and free from pain, although the mere absence of pain does not fully constitute the positive state of being comfortable. Rather, comfort is a broader construct which also incorporates the absence of other unpleasant physiological sensations (such as rough textures, extremes in temperature or excessive moisture) and the presence of highly subjective feelings (such as ease, support and contentment) [4, 5].

In addition to facilitating a general sense of wellbeing, the use of comfortable footwear is also considered to have a range of practical advantages, as it may facilitate physical activity [6], enhance sporting performance [7], and reduce the incidence of injury [8]. Therefore, identifying the footwear design, physiological and psychological factors which influence comfort could assist in the development and manufacture of improved footwear for a wide range of population groups, and potentially have both individual and societal benefits. Accordingly, the objective of this study was to provide a narrative synthesis of the research literature pertaining to footwear comfort, including definitions, measurement scales, footwear design features, and physiological and psychological factors.

## Methods

**Fig. 1:**
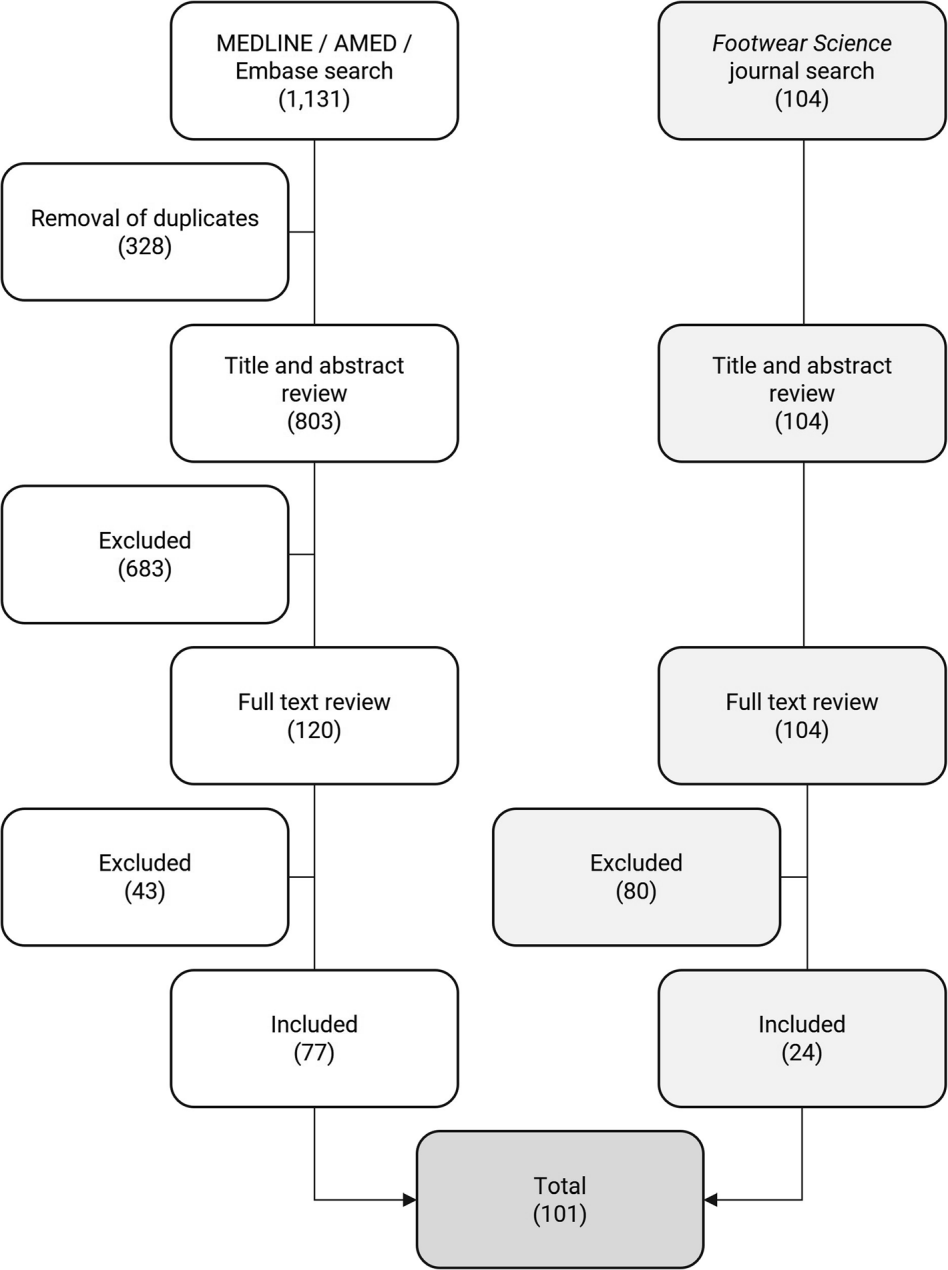
Flowchart of included papers

A systematic search was initially conducted in December 2020 and updated in August 2021. The Ovid platform was used to explore MEDLINE (1946 to present), AMED (1985 to present) and Embase (1974 to present) by applying the search string ((footwear or shoe*) and comfort*).mp, limited to human and English language papers. All study designs were considered. Articles addressing comfort of footwear and/or insoles were included, but studies on hosiery, cast walkers or ankle-foot orthoses were excluded. This search was supplemented by a title and abstract search of *Footwear Science*, as this journal is not indexed in MEDLINE, AMED or Embase. The Ovid search yielded 1131 documents, and after the removal of 328 duplicates, 803 documents were screened by title and abstract. Following title and abstract review there were 120 relevant documents, and after full-text screening 77 documents were included. The *Footwear Science* search identified 104 documents which was reduced to 24 after full-text review, giving a combined total of 101 documents [1, 3, 5, 7, 9–105] (see Fig. 1). The most relevant manuscripts were selected based on the predetermined subheadings of the review (definitions, measurement scales, footwear design features, and physiological and psychological factors). A narrative synthesis of the findings of these studies was then undertaken.

## Results

### Characteristics of included studies

Most studies were laboratory-based, repeated measures designs where comfort was measured under different footwear and/or insole conditions [14, 15, 18, 20–23, 25–30, 33, 35–39, 41, 43–48, 50–55, 57, 58, 60–71, 73–79, 81–87, 89, 90, 93, 97, 98, 100–105], but there were also 13 surveys [1, 3, 9–11, 16, 19, 24, 40, 56, 88, 92, 96], eight clinical trials [12, 34, 42, 49, 59, 94, 95, 99], three qualitative studies [13, 17, 91] and three reviews [5, 7, 72]. Sample size ranged from 5 to 1524, and primarily included healthy young adults [10, 14, 15, 17, 18, 22, 28, 31, 32, 35, 37, 43–46, 48, 50, 53, 54, 56, 58, 60, 64, 67, 74–76, 78, 79, 83, 88, 90, 101–104], but also children [71, 91], older people [1, 52, 63, 76], participants with medical conditions (such as diabetes [16, 27, 68, 105], rheumatoid arthritis [29], patellofemoral pain [73], plantar fasciitis [99], hallux valgus [93] and non-specific musculoskeletal disorders/symptoms [25, 95]), specific occupational groups (such as military personnel [42, 69, 85, 89], factory workers [9, 12], school teachers [11], kitchen staff [13], hospital staff [13], coal miners [40, 41] and police officers [94]) and sportspeople (such as runners [26, 30, 36, 39, 47, 49, 65, 66, 70, 77, 82, 84, 86, 98], basketball players [61, 62, 92, 100], soccer players [38, 87, 97], cyclists [20, 21], aerobic dancers [34], skiers [51], rugby players [59], people attending gymnasiums [3], badminton players [55] and tennis players [96]).

### Definitions of comfort

No studies provided a specific definition of comfort, although four studies were designed to explore how comfort is conceptualised. Alcantara et al. [10] developed a list of 74 adjectives related to footwear design and manufacture and asked 67 people to evaluate 36 shoes using these adjectives on a 5-point scale. Principal components analysis demonstrated that perception of casual footwear could be described on the basis of 20 independent concepts, two of which pertained to comfort. The first was characterised as ‘pure comfort’ and included the positive adjectives good fitting, soft, comfortable, flexible, light, relaxing, smooth, and the negative adjectives rough, hard, strong, heavy, rigid, and robust. The second was characterised as ‘thermal comfort’ and included the positive adjectives fresh, light, breathable, and the negative adjectives hot, heavy, and safe. Similar findings were reported in a qualitative study of footwear comfort perceptions of standing workers by Anderson et al. [13], who found that positive adjectives used were cushioning, arch support/contour, breathability/ventilation and negative adjectives used were hardness, heaviness and heat. A study of younger women’s perceptions of dress shoes identified ten criteria which differentiated between comfortable and uncomfortable shoes, the strongest being absence of pain, feeling, sound, and texture [17]. Finally, in a qualitative study of children, the adjectives soft and padding were most frequently used to describe comfortable shoes, while hard, tight, loose and heavy were used to describe uncomfortable shoes [91].

### Comfort measurement scales

A wide range of measurement tools have been used to quantify comfort, including simple dichotomous responses [11, 12, 29, 94], ranking footwear conditions in order of preference [14, 31–33, 36, 47, 48, 52, 60, 63–65, 76, 77, 79, 81], 4-point [42, 80], 5-point [37, 40, 45, 58, 70, 76, 97], 6-point [59], 7-point [47, 64, 79, 87], 9-point [57] and 12-point [41] Likert scales, 10-point numerical rating scales [43, 78], and 100 mm [15, 16, 18–21, 27, 28, 30, 35, 39, 48, 49, 53, 56, 63–65, 67, 68, 71, 73, 74, 77, 79, 82, 85, 89, 90, 93, 99, 104, 105], 150 mm [22, 25, 26, 36, 46, 50, 55, 61, 62, 66, 69, 75, 83, 84, 98, 100] and 170 mm [44] visual analog scales. The anchor statements indicating the lowest possible comfort score included ‘not comfortable at all’ [19–22, 25, 26, 30, 39, 49, 50, 53, 55, 56, 61–65, 73, 79, 82–85, 89, 93], ‘very uncomfortable’ [27, 28, 40, 41, 45, 46, 58, 68, 74, 78, 79, 98], ‘least comfortable’ [44, 76, 80, 90], ‘extremely uncomfortable’ [48, 59, 89], ‘not comfortable’ [71, 75], ‘not at all comfortable’ [15, 18], ‘not acceptable’ [37, 70], ‘totally disagree’ [10], ‘least comfortable imaginable’ [35], ‘not satisfactory’ [42], ‘very bad comfort’ [43], ‘very, very low’ [47], ‘minimum comfort’ [69], ‘maximal pain/discomfort’ [99], ‘not very comfortable’ [100], ‘completely uncomfortable’ [104], ‘extremely bad’ [57] and ‘unbearable discomfort’ [87]. The anchor statements indicating the highest possible comfort score included ‘most comfortable imaginable’ [19–22, 25, 26, 30, 35, 39, 46, 50, 53, 56, 61, 63, 65, 73, 79, 82–84], ‘very comfortable’ [18, 27, 28, 40, 41, 49, 58, 68, 71, 74, 75, 78, 79, 98, 100], ‘most comfortable’ [44, 55, 62, 76, 80, 85, 90], ‘extremely comfortable’ [48, 59, 87], ‘just right’ [37, 70], ‘totally agree’ [10], ‘very much’ [15], ‘excellent’ [42], ‘very good comfort’ [43], ‘not at all uncomfortable’ [45], ‘very, very high’ [47], ‘maximum comfort’ [69], ‘maximal comfortable’ [64], ‘no pain/discomfort’ [99], ‘completely comfortable’ [104], ‘most conceivable comfort’ [93] and ‘extremely good’ [57]. Most studies documented an overall comfort score for the whole foot/shoe, while others reported separate comfort scores for specific regions of the foot/shoe [19, 21, 22, 36, 57, 59, 64, 66, 71, 73, 75, 83, 87, 104]. The vast majority of tools considered comfort to be a unidimensional construct, although some incorporated additional perceptual components including in-shoe ‘climate’ [55], thermal comfort [51, 67, 103], dampness [67] and air permeability [67].

Seven studies specifically addressed the psychometric properties of comfort scales. Mündermann et al. [83] assessed the reliability of 150 mm visual analog scales documenting comfort pertaining to forefoot cushioning, heel cushioning, arch height, heel cup fit, shoe heel width, shoe forefoot width, and shoe length in runners wearing standardised running footwear with four inserts of differing hardness. Overall, intra-test repeatability was high (intraclass correlation coefficient [ICC] 0.80 and improved with four to six repeated sessions, although it was noted that some participants reported highly variable comfort ratings. A subsequent study by these authors demonstrated consistency of scores using this scale with repeated sessions over 3 weeks [84], and Lam et al. [62] confirmed the reliability of the same 150 mm visual analog scale in basketball players (ICCs from 0.61 to 0.80). Mills et al. [79] compared the reliability of documenting comfort (overall, cushioning of the forefoot, arch and heel, and support of the arch and heel) in participants wearing their usual footwear and four different inserts using 100 mm visual analog scales, 7-point Likert scales and ranking, and found that ranking was the most reliable measure, followed by the visual analog scales and Likert scales. Similarly, Lindorfer et al. [64] assessed 30 runners over six repeated sessions, and found that ranking provided the highest reliability (Pearson’s *r* = 0.07), followed by a 100 mm visual analog scale (*r* = 0.67) and 7-point Likert scale (*r* = 0.63). More recently, Bishop et al. [19] reported a detailed psychometric evaluation of a new running shoe comfort assessment tool incorporating four components measured with a 100 mm visual analog scale (heel cushioning, forefoot cushioning, shoe stability, forefoot flexibility and an overall comfort score). Reliability of the overall score was excellent (ICC 0.88) and good for each of the component scores (ICC > 0.70).

In contrast to these positive findings, Hoerzer et al. [50] examined intra-rater reliability of 150 mm visual analog scales and dichotomous (yes/no) ratings of insole comfort, and found that less than a third of participants provided reliable scores across the two sessions. These findings suggest that psychological factors, such as mood, may influence the perception of comfort and that documenting a mean score across multiple sessions may be necessary to obtain acceptable reliability. Furthermore, a recent systematic review of comfort scales by Matthias et al. [72] demonstrated that few studies explicitly evaluated validity, and many exhibited methodological bias, such as lack of participant and assessor blinding.

### Footwear design features associated with comfort

#### Fit

Three studies explored the effect of footwear fit on comfort. Miller et al. [78] evaluated associations between foot anthropometric measurements and comfort while wearing three different running shoes, and found that a range of measurements (particularly related to shoe fit in the forefoot and toes) influenced comfort perceptions. However, these associations varied across the three styles, suggesting that fit may differentially influence comfort depending on other characteristics of the shoe. In the second study, Branthwaite et al. [22] assessed the effect of toe-box constriction on comfort by comparing three toe-box shapes in ballet pumps (round, square and pointed). Although there was no difference in comfort scores, none of the shoes were considered to be comfortable. Most recently, Matthias et al. [71] assessed comfort ratings of children aged 8 to 12 years while wearing school shoes that were appropriately fitted for size, one size too large, and one size too small. The fitted shoes were rated as the most comfortable overall, while the smaller size was rated as too tight in the heel and toe regions.

### Midsole cushioning

Eight studies evaluated perceived comfort while wearing footwear that varied according to midsole cushioning, including running shoes [39, 60, 78, 90, 98], basketball shoes [61], casual shoes [63] and military boots [85]. All but one study [63] reported that participants found the footwear with softer midsole materials to be more comfortable, although Sterzing et al. [95] also demonstrated that the use of harder materials under the forefoot did not negatively affect comfort provided that the material under the rearfoot was soft. However, documentation of midsole cushioning across these studies was inconsistent, with some studies using no objective measures [60, 61, 78] and others reporting either density (which ranged from 0.15 to 0.24 g/cm^3^) [39, 90] or hardness (which ranged from Shore A 25 to 66) [63, 85, 90]. Furthermore, only three of these studies [61, 63, 98] specifically manipulated midsole cushioning while controlling for other shoe characteristics, making it difficult to attribute differences in comfort to cushioning alone.

### Heel elevation

Nine studies examined the effects of heel elevation on comfort perception while wearing high-heels [28, 53, 54, 56, 101], casual shoes [75, 76] and running shoes [23, 82]. In the two running shoe studies, heel elevations (i.e., heel-toe drop) ranged from 0 to 15 mm, but no differences in comfort were found during treadmill walking [23] or running [82]. In contrast, significant reductions in comfort were consistently reported in each of the studies examining high-heels [28, 53, 54, 56] and casual shoes [75, 76] when heel height was increased by between 45 and 76 mm. However, comfort perception was higher in participants who were habituated to wearing high heels [28], and three studies reported that the discomfort associated with heel elevation could be partly ameliorated by the use of heel cups [56] and arch supports [53, 54, 56]. An interaction between heel height and the sagittal plane angle of the heel seat (‘wedge angle’) was also reported by Witana et al. [101], who found that comfort while wearing high heels could be optimised by selecting the most appropriate wedge angle for a corresponding heel height.

### Weight

Five studies evaluated the influence of shoe weight on perceived comfort in military boots [85, 89], safety footwear [18], running shoes [60] and basketball shoes [62]. However, only three studies provided objective measurements of shoe weight (ranging from 335 to 800 g) [62, 85, 89] and none controlled for other shoe characteristics. Nevertheless, all reported that the lightest shoe was perceived to be the most comfortable. In addition to the absolute weight of the shoe, the distribution of mass may also be important. Chiu et al. [33] added weights to different locations of casual canvas shoes while keeping the total weight constant, and found that most participants preferred rear-weighted shoes and perceived them to be lighter than when the weights were added distally.

### Sole flexibility/bending stiffness

Four studies examined the effect of sole flexibility on comfort: two in running shoes [37, 78], one in coal mining boots [41], and one in people with diabetes [105]. In running, comfort related to sole flexibility may depend on running speed. Miller et al. [78] compared comfort while standing, walking and running in participants wearing three different commercially-available running shoes, and found that although comfort ratings differed according to the activity, the most comfortable shoe on average was the most flexible. However, Day et al. [37] tested participants in shoes with and without carbon fibre plates and found that participants preferred the standard shoe when running at 14 km/h, but the stiffer shoe at 17 km/h. The coal mining boot study assessed comfort when coal miners wore two standard boots (one with a stiff shaft and one with a flexible shaft), and each boot was then modified to create a more flexible sole by cutting slits across the sole at the level of the metatarsophalangeal joints. Although most participants preferred the boot with a flexible shaft combined with a stiff (unmodified) sole, there was large variability in the comfort scores and no significant effect among the different boot types [41]. Finally, Zwaferink et al. [105] found that adding a 3 mm carbon-fibre stiffening insert to extra-depth shoes in people at risk of foot ulceration resulted in lower plantar pressures, but had no detrimental effect on comfort.

### Midsole geometry

Seven studies evaluated the influence of various aspects of midsole geometry on comfort, including offloading/rocker-sole footwear (in healthy individuals [43, 66], older people [52], people with diabetes [27, 68] and people with rheumatoid arthritis [29]), and the application of lateral wedges to footwear for the treatment of knee osteoarthritis [44]. In the offloading footwear studies, rocker-sole shoes were perceived to be more comfortable than standard footwear [29] or forefoot offloading shoes (i.e., shoes with no ground contact at the forefoot designed to avoid toe-off) [27, 43], and rigid rocker-sole shoes more comfortable than semirigid rocker-sole shoes [68]. However, no substantial differences in comfort were found across three different heel curvature designs (short-parallel, long-parallel and oblique) in running shoes [66], adding foam to the plantar midfoot region of the outersole [52], or following the addition of a small (2 degree) lateral heel wedge designed to alter knee joint moments when walking [44].

### Outsole geometry

Three studies evaluated the effect of outsole geometry on comfort [38, 59, 87]. In soccer boots, de Clerq et al. [38] assessed the effect of various stud configurations on comfort when performing cutting manoeuvres, and found that the sole design with the least number of studs was the most comfortable. Kryger et al. [87] compared two soccer boot designs that varied according to stud shape, upper material and boot mass, and found that the most comfortable boot generated lower pressures under the first and fifth metatarsal heads. Similarly, in rugby, Kinchington et al. found that hybrid turf shoes were more comfortable than studded boots [59].

### Lacing

Two studies evaluated the effect of lacing on comfort. Dobson et al. [40] reported lace-up boots to be more comfortable than slip-on boots in coal miners, while Hagen et al. [58] compared different styles of lacing in seven-eyelet running shoes, and found that comfort varied according to the number of eyelets laced and how tightly the laces were tied. The most comfortable technique involved the use of three eyelets laced while keeping the upper two eyelets unlaced, although this was perceived to be less stable and was associated with higher pronation velocity while running compared to tightly lacing all eyelets.

### Upper material

Four studies demonstrated that shoes with more compliant upper materials may be preferable. Jordan et al. [58] compared three styles of casual footwear and found that shoes generating lower pressures on the dorsum of the foot were perceived as more comfortable, while Melvin et al. [75] found that shoes constructed from soft suede were more comfortable than shoes constructed from a stiffer leather upper. In soccer boots, Sterzing et al. [97] found that despite two models having identical stud configurations, the model with the softer heel counter was perceived to be more comfortable. Finally, Saeedi et al. [93] evaluated people with hallux valgus wearing their own shoes, shoes with a round toe-box and shoes with a stretchable fabric upper, and found that the shoes with the stretchable upper generated lower toe pressures and were perceived to be the most comfortable.

### Shoe microclimate

Four studies evaluated the influence of shoe microclimate (i.e., temperature, moisture and ventilation characteristics) on comfort, generally in the context of footwear worn in cold environments (such as trekking boots [15], safety boots [45] and ski boots [51]), but also in sandals used in combination with footwarmers indoors [103]. The findings of these studies were inconsistent, in that higher temperatures were found to be associated with improved comfort perceptions in ski boots [51], when adding insulation and toecaps to safety boots [45] and in indoor sandals [103], but lower temperatures were found to be more comfortable in trekking boots [15]. Likely explanations for this inconsistency are the range of ambient temperatures each study was performed in (which varied from − 6.8 to 23.3 °C) and different methods for measuring in-shoe temperature. The role of moisture and ventilation has been less studied and is inherently difficult to delineate from temperature effects. However, the study of trekking boots by Arezes et al. [15] found moisture retention to be of secondary importance to temperature in determining comfort levels.

### Insoles

Twenty-six studies have been conducted to assess the effects of insoles on footwear comfort. However, it is difficult to draw clear conclusions from the available literature due to the variation in populations studied and wide range of insole designs used. Broadly, the evidence suggests that the addition of insoles improves footwear comfort in casual footwear [95], factory footwear [12], running shoes [25, 26, 49, 84], high heels [53, 54] and police boots [94]. However, no significant improvements in comfort have been reported when adding flat cushioning insoles to walking shoes [74] or running shoes [86], or contoured insoles to cycling shoes [20]. Furthermore, the effect of insoles on footwear comfort is influenced by the fit of the shoe, as it has been observed that insoles may decrease comfort if they make the shoe too tight [34].

Studies comparing different insole designs have generally found that softer, more flexible insoles are perceived as more comfortable (in casual footwear [99], running shoes [31, 67, 80] and military footwear [42, 69]). However, comfort perceptions related to insole hardness may vary according to an individual’s occupation, as Anderson et al. [14] have reported that people standing for long periods at work prefer soft materials under the heel and forefoot but firmer materials under the arch. The influence of insole shape is uncertain, with studies reporting flat insoles to be more comfortable than contoured [48], contoured more comfortable than flat [69, 100], or no difference between the two [73]. Furthermore, the effect of insole customisation is unclear. Fully customised orthoses have been reported to be more comfortable than semi-customised insoles in runners [36], while reductions in comfort have been observed with the addition of anterior wedges when performing a load lifting task [46] and lateral forefoot posting when cycling [21].

### Wear time

One study assessed the effect of repeated wear on comfort while wearing badminton shoes [55]. Badminton players performed direction change manoeuvres while wearing new shoes and then the same shoes after 96 h of wear (6, 2-h training sessions per week for 8 weeks). The worn shoes were perceived to be significantly less comfortable in relation to in-shoe climate, medio-lateral stability, and overall fit, although performance in direction change manoeuvres was not adversely affected.

### Physiological and psychological factors associated with comfort

#### Sex

Two studies assessed sex differences in comfort perception in running shoes, with inconsistent findings [57, 60]. Kong et al. [60] instructed healthy men and women to walk and run in three types of footwear (cushioning, lightweight and stability) and asked them to select the model that they found most comfortable. No differences were noted between walking and running, but women were four times more likely to select the lightweight shoe compared to men, which the authors attributed to women weighing less and therefore preferring a shoe with less metabolic energy cost. In contrast, Isherwood et al. [57] analysed running biomechanics in men and women wearing the same running shoe model, and despite noting several sex-related differences in kinematics and kinetics, found no difference in perception of cushioning, stability, or overall comfort.

#### Foot-related factors

Four studies have evaluated associations between foot characteristics and comfort, specifically addressing foot alignment [14, 78, 104] and tactile sensitivity [81]. In relation to arch height, Zifchock et al. [104] compared comfort ratings while wearing custom and semi-custom orthoses, and found that participants with high arches reported greater arch and heel comfort in the semi-custom device which provided less rearfoot control when walking, while Anderson et al. [14] assessed perceptions of nine different insoles which varied according to the hardness of the heel, midfoot and forefoot in participants working in occupations that require prolonged standing, and found that those with lower arched feet preferred insoles with harder material in the midfoot. Miller et al. [78] compared comfort perceptions when walking and running in three shoes that varied in relation to stiffness, cushioning and shape, and found that heel eversion angle was negatively associated with comfort in the stiffer, harder soled shoe. Finally, Mills et al. [81] compared minimalist and cushioned shoes during running and found that individuals who ranked the cushioned shoe as most comfortable demonstrated higher sensitivity to mechanical pain at their heel and midfoot.

#### Perceptual factors

Three studies explored the effects of perceptual factors on footwear comfort. Chamb et al. [30] evaluated running biomechanics on a treadmill under two footwear conditions (shoe A and B). Identical running shoes were used in both conditions, but shoe B was described to be the “latest model designed to maximize comfort” and more expensive than shoe A. Although no differences in running biomechanics were evident, runners rated shoe B as significantly more comfortable than shoe A, demonstrating that comfort ratings can be biased by marketing and perceived quality related to cost. However, runners’ perceptions of comfort do not appear to be strongly associated with *actual* cost of shoes. Clinghan et al. [35] measured plantar pressures, comfort scores and perceived cost of shoes at three price ranges, but found no significant associations, suggesting that comfort is highly subjective and based on individual preferences. Finally, in basketball players, Wang et al. [100] evaluated comfort perceptions when performing drop landings while wearing insoles that differed according to colour and contour, and found that red insoles were perceived to be more comfortable than white insoles with the same contour.

## Discussion

**Fig. 2:**
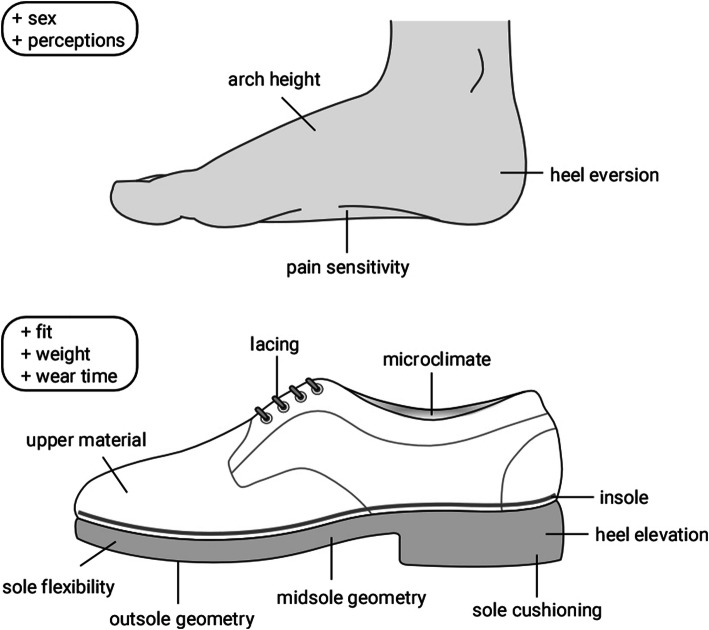
Summary of factors associated with footwear comfort

The objective of this study was to provide a summary and critique of the research literature pertaining to footwear comfort. Overall, the available evidence is highly fragmented and incorporates a wide range of study designs, participants, and assessment approaches, making it challenging to draw strong conclusions or implications for clinical practice. It is evident that footwear comfort is a complex and multifaceted concept, that perceptions of comfort are highly subjective, and that comfort is influenced not only by structural and functional aspects of shoe design, but also anatomical and physiological differences between individuals and the unique requirements of the occupational or sporting activity being performed. Nevertheless, there is sufficient uniformity in key findings to provide some broad recommendations as to how comfort should be assessed and what constitutes a comfortable shoe. A summary of the key factors influencing comfort are shown in Fig. 2.

Somewhat surprisingly, no studies specifically defined comfort, and a wide range of assessment scales were used. The anchor statements were also highly variable and conceptually inconsistent. For example, some scales considered the *absence* of comfort as the worst state (with ‘not comfortable’ as the anchor statement), whereas other scales were bidirectional and used anchors such as ‘extremely uncomfortable’ for the lowest possible scores. From a psychometric perspective, it is unlikely that ‘not comfortable’ and ‘extremely uncomfortable’ represent the same construct, so making comparisons between studies using different scales is problematic. Nevertheless, the most widely used overall comfort tools (100 mm or 150 mm visual analog scales) demonstrated moderate to high reliability and could therefore be recommended for future use. However, as suggested by Matthias et al. [72], it would be advisable to evaluate reliability within each individual study’s sample population, and to conceal the external appearance of the shoe to avoid bias introduced by participant’s perceptions of footwear aesthetics.

Despite the wide range of occupational and sporting groups evaluated in these studies and their different footwear requirements, it would appear that there are some generic design principles that constitute a comfortable shoe. First, a comfortable shoe is one that fits the foot appropriately, although it needs to be recognised that some very specific situations may require excessively tight shoes (e.g., ballet [106] and rock-climbing [107]). Second, softer and more compliant materials are generally regarded as being more comfortable than harder materials in the upper, midsole and insole. Third, with the exception of individuals who have become habituated to high heels over long periods of use [28], lower heel elevation is generally associated with greater perceived comfort. Fourth, the available evidence suggests that lightweight shoes are generally preferred over heavier shoes. Finally, curved rocker-soles appear to be beneficial for comfort compared to flat soles in a range of population groups.

Less consistency was observed for sole flexibility, in-shoe temperature and insoles. The most likely explanation for this is that the effects of these features on comfort are more specific to the population, setting and task. For example, while runners generally prefer a flexible sole, coal miners prefer a more rigid sole, presumably as this facilitates more comfortable ambulation on uneven or unstable terrain. Similarly, while relatively lower in-shoe temperatures are generally perceived as more comfortable under routine climatic conditions (generally temperatures of 5 to 25 °C) [5], higher in-shoe temperatures are preferred in the context of lower ambient temperatures, such as when wearing trekking or ski boots. Finally, although the evidence broadly indicates that the addition of soft insoles to shoes generally improves comfort, the wide array of insole designs (materials, contour, posting and wedging) makes it difficult to reach definitive conclusions.

Few studies examined how comfort ratings are influenced by the interaction between footwear and individual characteristics of the wearer. Given that comfort is a complex neurophysiological and psychological construct, it is likely that variability in an individual’s body mass, skeletal alignment, joint range of motion, gait pattern, tactile sensitivity, pain perceptions and aesthetic preferences will influence whether they perceive a particular shoe to be comfortable. While the available evidence suggests that foot structure, function and pain sensitivity may influence insole contour and sole hardness preferences, further research is required to optimise the identification of footwear features that are most suitable for an individual’s anatomical and physiological characteristics.

It is worth noting that while comfort is one of the key considerations when selecting footwear, other factors, such as performance and injury risk, also need to be considered and that these requirements are not necessarily compatible. For example, in running shoes, leaving the top two eyelets unlaced is perceived as the most comfortable but also least stable lacing technique, and results in higher pronation velocity which may increase the risk of injury [58]. Similarly, while older people may find shoes with softer midsole materials to be more comfortable, there is evidence that excessively soft midsoles may be detrimental to balance and therefore increase the risk of falls [108]. Clearly, comfort is not the only requirement of footwear, and in some circumstances, comfort may need to be compromised to ensure other needs are met. This is particularly true for some types of occupational footwear, where important safety features (such as steel toe caps, rigid upper materials, and non-slip soles) may be detrimental to comfort but are essential for the prevention of workplace injury.

The findings of this review need to be interpreted in the context of several key limitations in the available literature. Firstly, a wide range of comfort measurement tools were used, so comparisons between studies is inherently problematic. Secondly, few studies specifically manipulated individual footwear design features while controlling for other characteristics, making it difficult to attribute differences in comfort to each individual feature. Finally, although we were able to draw some general conclusions regarding factors that influence comfort, it is likely that these factors influence comfort in different ways depending on the specific requirements of the setting and the activity being performed.

In summary, this paper has provided an overview of how footwear comfort is conceptualised and evaluated and has examined the footwear design features and individual characteristics that influence the perception of footwear comfort. Although the literature is fragmented and often inconsistent, it can be concluded that (i) simple visual analog scales may provide a reliable (albeit unidimensional) assessment of comfort, (ii) well-fitted, lightweight shoes with soft midsoles and curved rocker-soles are generally perceived to be most comfortable, and (iii) the influence of sole flexibility, in-shoe temperature and insoles is less clear and likely to be more specific to the population, setting and task. Suggested improvements and directions for future research include (i) specifically manipulating individual design features while controlling for other shoe characteristics, (ii) exploring the influence of shoe microclimate in greater detail, and (iii) examining the interaction between footwear features and individual physiological attributes.

## Data Availability

Not applicable.
